# The multifaceted view of heart problem in Duchenne muscular dystrophy

**DOI:** 10.1007/s00018-021-03862-2

**Published:** 2021-06-06

**Authors:** Urszula Florczyk-Soluch, Katarzyna Polak, Józef Dulak

**Affiliations:** Department of Medical Biotechnology, Faculty of Biochemistry, Biophysics and Biotechnology, Jagiellonian University, Kraków, Poland

**Keywords:** *DMD* mutations, Non-ischemic cardiomyopathies, Heart failure, Supraventricular and ventricular arrhythmias, Fibrosis, Hypertrophy

## Abstract

Dystrophin is a large protein serving as local scaffolding repetitively bridging cytoskeleton and the outside of striated muscle cell. As such dystrophin is a critical brick primarily in dystrophin-associated protein complex (DAGC) and in a larger submembranous unit, costamere. Accordingly, the lack of functional dystrophin laying at the root of Duchenne muscular dystrophy (DMD) drives sarcolemma instability. From this point on, the cascade inevitably leading to the death of myocyte begins. In cardiomyocytes, intracellular calcium overload and related mitochondrial-mediated cell death mainly contribute to myocardial dysfunction and dilation while other protein dysregulation and/or mislocalization may affect electrical conduction system and favor arrhythmogenesis. Although clinically DMD manifests as progressive muscle weakness and skeletal muscle symptoms define characteristic of DMD, it is the heart problem the biggest challenge that most often develop in the form of dilated cardiomyopathy (DCM). Current standards of treatment and recent progress in respiratory care, introduced in most settings in the 1990s, have improved quality of life and median life expectancy to 4th decade of patient’s age. At the same time, cardiac causes of death related to DMD increases. Despite preventive and palliative cardiac treatments available, the prognoses remain poor. Direct therapeutic targeting of dystrophin deficiency is critical, however, hindered by the large size of the dystrophin cDNA and/or stochastic, often extensive genetic changes in *DMD* gene. The correlation between cardiac involvement and mutations affecting specific dystrophin isoforms, may provide a mutation-specific cardiac management and novel therapeutic approaches for patients with CM. Nonetheless, the successful cardiac treatment poses a big challenge and may require combined therapy to combat dystrophin deficiency and its after-effects (critical in DMD pathogenesis). This review locates the multifaceted heart problem in the course of DMD, balancing the insights into basic science, translational efforts and clinical manifestation of dystrophic heart disease.

## Introduction

Duchenne muscular dystrophy (DMD) is one of the most severe and devastating types of inherited muscular dystrophies which remains incurable despite the extensive investigation. The estimates of DMD overall prevalence and birth prevalence diverge depending on the study design (e.g. study population), ranging from 0.9 to 16.8 per 100 000 (1 per–111,000 to 1 per–6000) males and from 1.5 to 28.2 per 100,000 (1 per–67,000 to 1 per–3500) live male births, respectively [1]. With no geographic exclusion criteria, the pooled global birth prevalence of DMD is 19.8 cases per 100,000 (1 per 5000) live male births [1].

DMD is determined by the lack of dystrophin due to mutation in dystrophin gene (*DMD*) and reflected by loss of ambulation at or before age 12 [2]. In skeletal myofibers and cardiomyocytes dystrophin localizes in cortical cytoskeleton being crucial component of the dystrophin-associated glycoprotein complex (DAGC) and a part of a larger unit, costamere [3, 4]. As such it is fundamental to maintain myocyte integrity mediating the contact of the cell external environment with cytoskeleton and preventing contraction-induced damage. Accordingly, the lack of functional dystrophin causes mechanical instability and disruption of muscle membrane, that brings about clinical features of progressive muscle weakness and deterioration in motor performance in affected boys. At the onset, in the first decade of children’s life the waddling gait, lordotic posture and calf hypertrophy are presented following by difficulties in walking, and eventually wheelchair requirement [3].

Current standards of DMD management (including glucocorticoid treatment or invasive and non-invasive ventilation) have improved quality of life and median life expectancy to 4th decade of age (21–40 years; recent data from 2662 patients from 12 countries) [5, 6]. Permanent ventilation is often required around 23 years old to prevent respiratory failure [7]. Without ventilatory support patients died in 2^nd^ or 3rd decade of life (at 14–27 years; 2662 patients) [5, 7, 8].

The progression of heart problems is not correlated with the extent of skeletal and respiratory muscle involvement, while confers an alternative cause of death related to DMD [7]. Till 1980s, the respiratory causes of death were dominant over cardiac ones [8]. Recent progress in respiratory care, introduced in most settings in the 1990s [5], have contributed to a proportionate increase of cardiac causes of death [9, 10].

The first incidences of cardiac problems arising from the absence of dystrophin in cardiomyocytes, may begin by the age of 6 years and most often develop in the form of dilated cardiomyopathy (DCM), turning into end-stage heart failure (HF) along with associated supraventricular and ventricular arrhythmias [9, 11]. Clinical signs of HF can be undetected due to musculoskeletal limitations, masked due to poor mobility and low exercise capability related to skeletal muscle weakness or missed due to the use of nocturnal mechanical ventilation [3, 9]. This implies the need of active diagnosis, which despite the extensive use of advanced imaging techniques, is still to be refined. Current treatment for heart disease in DMD, although silences the symptoms and retards the disease progression, cannot reverse poor prognoses for patients with DMD.

## The roots of DMD

### Muscle-specific Dp427m among other dystrophin isoforms

**Fig. 1:**
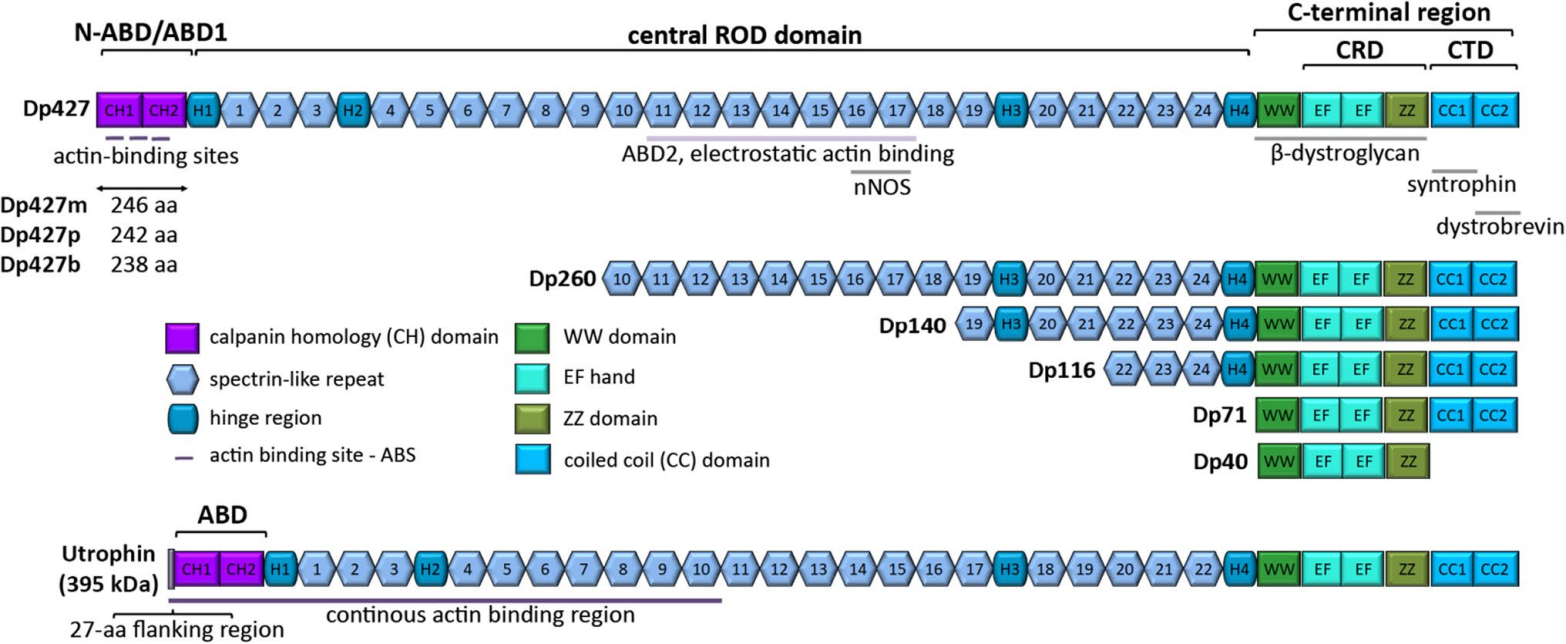
Dystrophin structure and isoforms. Three full-length (427 kDa) dystrophin isoforms, Dp427b, Dp427m and Dp427p, differ only in N-terminal flanking regions. As Dp427m, shorter variants have preserved C-terminal region, with WW domain, two EF hands and ZZ domain, enabling the β-dystroglycan binding and subsarcolemmal localization. Utrophin, an autosomal paralogue with high homology to dystrophin, lacks two spectrin-like (SR) repeats (15 and 19) and presents distinct mode of actin binding. Dystrophin binds actin laterally along the filament via joint action of 2 low-affinity binding regions, actin-binding domains, ABD1 and ABD2, bridging more actin monomers than utrophin, which interacts with actin via one continuous binding site

*DMD* gene comprises 79 exons on the X chromosome (Xp21) and contains at least 7 tissue-specific promoters and 2 polyA-addition sites, producing several isoforms with multiple splice variants [12]. Three upstream (5′) promoters, the brain (B), muscle (M) and Purkinje (P), generate full-length transcripts starting at unique first exons, which give rise to full-length (427 kDa) dystrophin isoforms: Dp427b, Dp427m and Dp427p, respectively. Dp427b is expressed mainly in hippocampal and cortical neurons, Dp427p in cerebellar Purkinje cells, while Dp427m in skeletal and cardiac myocytes and in glial cells at very low level. Shorter transcripts can be produced due to the action of specific internal promoters within intron 29 (R), 44 (B3), 55 (S), and 62 (G), generating shorter isoforms, respectively: retinal (Dp260), brain (Dp140), Schwann cell (Dp116) and general (ubiquitous) (Dp71). The shortest known isoform, Dp40 (expressed in hippocampal neurons), shares a first exon with Dp71 but both use alternative polyadenylation sites [3, 12] (Fig. 1).

**Fig. 2:**
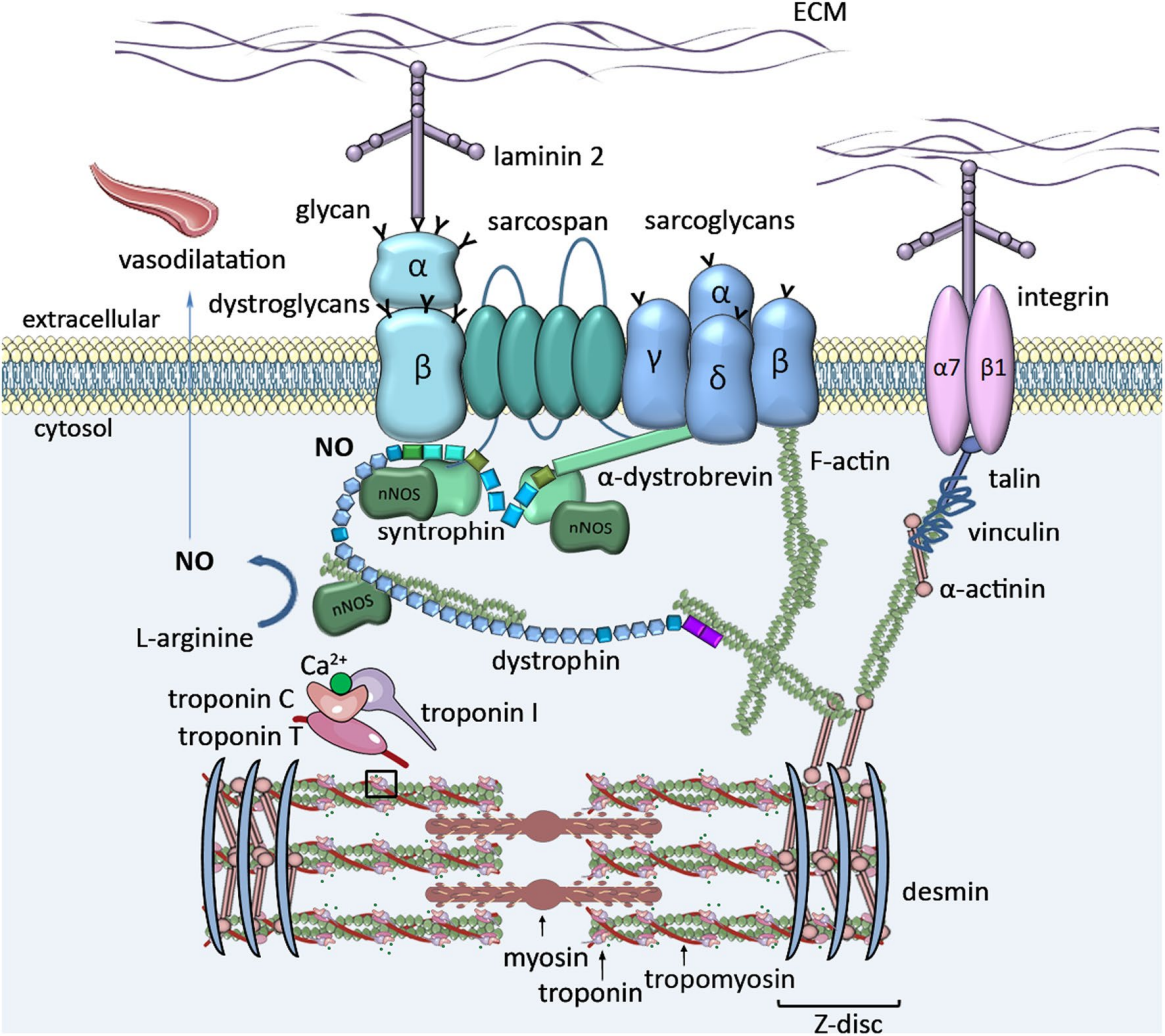
DAGC in striated muscle cell. Dystrophin is a critical brick in dystrophin-associated protein complex (DAGC) that together with integrin/talin/vinculin complex constitutes a main part of larger submembranous unit, costamere. Costamers span the sarcolemma of striated muscle repetitively joining sarcomeres to the sarcolemma. *ECM* extracellular matrix, *NO* nitric oxide, *nNOS* neuronal nitric oxide synthase

The full-length dystrophin (427 kDa) is a rod-shaped intracellular protein (Fig. 1). The N-terminal actin-binding domain (N-ABD or ABD1) is composed of two calponin-homology (CH) domains in tandem. CH tandem interacts with filamentous actin (F-actin) through three actin-binding surfaces (ABS1, ABS2, and ABS3). CH1 domain is essential for actin binding, CH2 provides stability to ABD1 [13]. The C-terminal region consists of several protein–protein interaction motifs. The first motifs, WW domain and two EF hands together bind β-dystroglycan. Subsequent ZZ domain enhances this binding. Due to the sequence composition, EF and ZZ domains are called the cysteine-rich domain (CRD). At the C terminus, aptly called C-terminal domain (CTD) is formed by two-coiled coil (CC) domains which enable binding of syntrophin (to CC1) and dystrobrevin, the shortened dystrophin homolog (to CC2) [14]. The N-terminal and C-terminal regions are separated by a long central rod domain, composed of 24 structurally similar spectrin-like repeats (SR) and 4 hinge regions. A second actin-binding domain (ABD2) is found between SR 11 and 17. Via SR16-17 or indirectly via syntrophin, dystrophin may anchor neuronal NOS (nNOS) to the sarcolemma [14, 15] (Figs. 1,2).

**Fig. 3:**
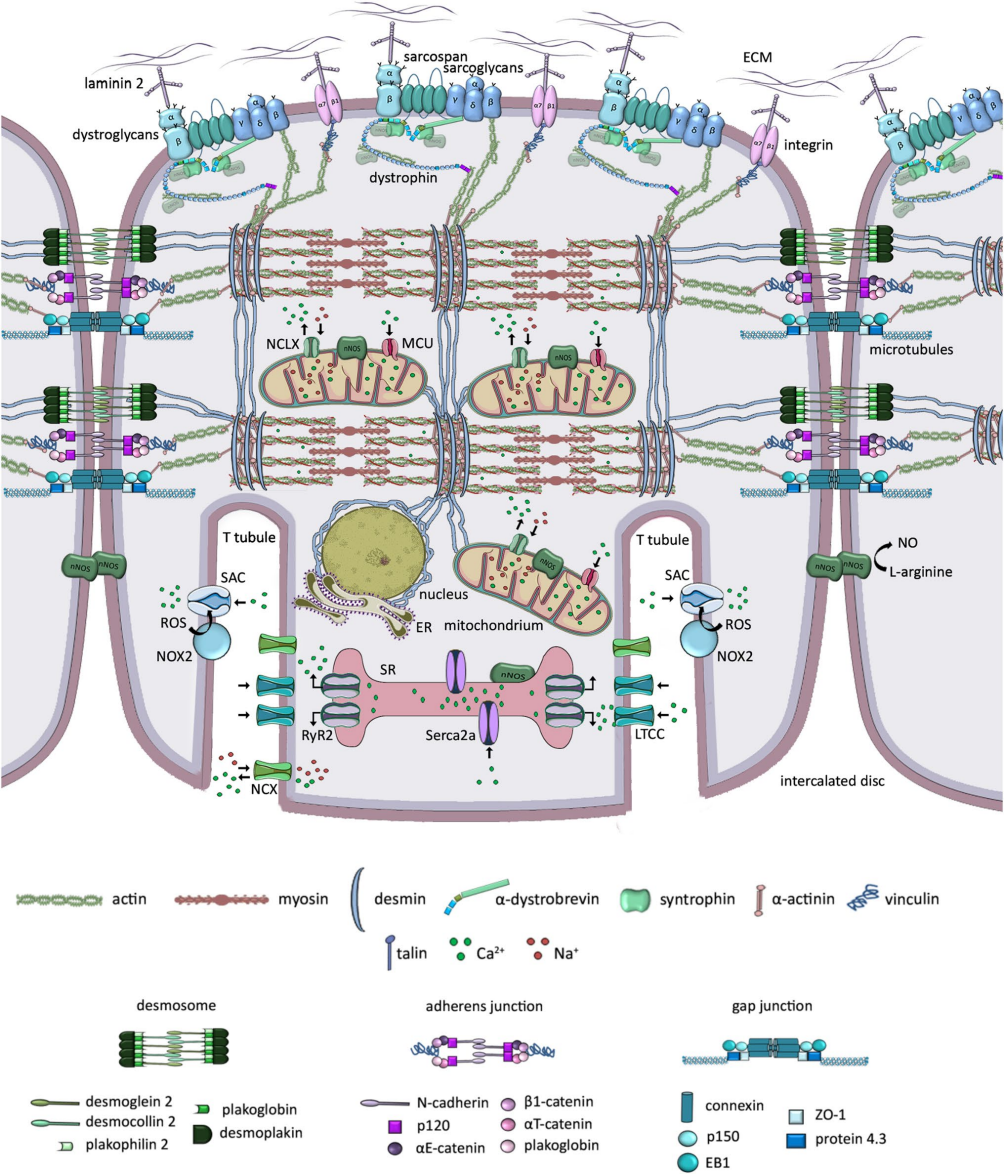
Healthy cardiomyocyte. Descirption in the text. *ER* endoplasmic reticulum, *LTCC* L-type calcium channel, *MCU* mitochondrial uniporter, *NCLX* Na^+^/Li^+^/Ca^2+^ exchanger, *NCX* Na^+^/Ca^2+^exchanger, *nNOS* neuronal nitric oxide synthases, *NO* nitric oxide, *NOX* NADPH oxidase, *ROS* reactive oxygen species, *RyR* ryanodine receptor, *SAC* stretch-activated channel, *Serca* sarco/endoplasmic reticulum Ca^2+^-ATPase, *SR* sarcoplasmic reticulum

The activity of nNOS results in the production of nitric oxide (NO), a key signaling molecule for contracting muscles, controlling blood flow and exercise-induced glucose uptake, Ca2 + handling, mitochondrial biogenesis and gene expression [16]. In striated muscles, nNOSµ splice variant is an essential NOS isoform [17]. In contrast to skeletal myofibers, nNOS does not colocalize with dystrophin or utrophin (see below) at sarcolemma of wild-type mouse cardiomyocytes [18]. In the heart, nNOS is detected on sarcoplasmic reticulum membrane [19], mitochondria [20] and the intercalated discs of cardiomyocytes (Fig. 3) [17].

The ABD1 of full-length dystrophin isoforms (Dp427m, Dp427p, Dp427b), composed of 246, 242, and 238 amino acid residues, respectively, differ only in N-terminal flanking region before the ABS1 [13]. Such minor variations determine much higher thermodynamic stability of tandem CH domain of Dp427m, but show lower F-actin-binding affinity compared to Dp427p and Dp427b [13]. All shorter isoforms lack the N-terminal domain and a part (Dp260-with preserved actin and nNOS-binding regions, Dp140, Dp116) or the whole (Dp71, Dp40) central rod domain (Fig. 1) [21].

Each isoform has selective spatial and temporal expression pattern. The presence of the unique first exons (i.e. not included in any other isoform) of Dp427p, Dp427b, Dp427m, Dp260, Dp140, Dp116 and the shared first exon of Dp71 and Dp40 can be used to distinguish the level of different isoforms [12].

Dp427m is predominant, but not secluded dystrophin isoform in human skeletal muscles and myocardium (Table 1). Dp427b can be found in fetal heart and both fetal and adult skeletal muscles [22, 23]. In the adult heart, the brain isoform is confined to atrial cardiomyocytes, but not expressed in ventricles or conduction system structures, unlike Dp427m that is present in all cardiac regions [24]. In fetal myocardium, both muscle and brain isoforms are detected from the early stages of development, whereas in skeletal muscle, the appearance of Dp427m precedes Dp427b [22]. Dp427p is undetectable in human fetal heart [22]. Little transcriptional activity of P promoter can be found in adult heart and fetal skeletal muscle, in contrast to significant expression of P isoform detected in adult skeletal muscle [22, 25]. Among shorter isoforms, Dp116 transcript was identified in both adult cardiac and skeletal muscle [26], whereas Dp7l expression only in fetal and adult heart byopsies [27]. Considering above, dystrophin isoforms should not be considered as tissue specific, but rather tissue selective.

**Table 1:** The expression pattern (protein or transcript) of dystrophin isoform in human cardiac and skeletal muscles

Isoform	Skeletal muscle		Heart	
	Fetal	Adult	Fetal	Adult
Dp 427 m	+	+	+	+
Dp 427b	+	+	+	+ (only atria)
Dp 427p	+	+	–	+
Dp 260	?	?	?	?
Dp 140	?	?	?	?
Dp 116	?	+	?	+
Dp 71	?	–	+	+
Dp 40	?	?	?	?

In addition to dystrophin isoforms, an autosomal functional paralogue of dystrophin exists, known as utrophin (395 kDa). The sequences of utrophin and dystrophin are homologous throughout their length, with utrophin lacking two SR (15 and 19). That change precludes nNOS anchorage to the sarcolemma via utrophin [15]. Both, utrophin and dystrophin, bind actin with similar affinities and both prevent actin disassembly. However, the modes of interaction with actin are distinct. Dystrophin binds actin laterally along the filament via joint action of two low-affinity binding regions, ABD1 and ABD2, bridging more actin monomers than utrophin, which interacts with actin via one continuous binding site. The latter is composed of the N-terminal domain (ABD) with additional 27-residue flanking region before its CH1 domain, and a string of the first 10 SR of the rod domain, which augments the affinity and capacity of ABD for actin [28, 29].

In fetal muscle, utrophin is ubiquitously expressed and distributed at the membrane being progressively replaced by dystrophin during late stages of embryonic development. During that time, both proteins may co-localize at the sarcolemma of the same muscle fiber. In adult muscle, utrophin is restricted to the myotendinous (MTJ) and neuromuscular junctions (NMJ) and blood vessels, while dystrophin is already present throughout the sarcolemma [30].

### Dystrophin—an important brick in costamere

In striated muscles dystrophin interacts with several proteins to assemble DAGC, distributed throughout the sarcolemma (Fig. 2). The complex is built by intracellular (α1-syntrophin and β1-syntrophin, α-dystrobrevin, and nNOS), transmembrane (β-dystroglycan, α-sarcoglycan, β-sarcoglycan, γ-sarcoglycan, and δ-sarcoglycan, and sarcospan) and extracellular (α-dystroglycan and laminin-2) components [31]. Little is known about DAGC in non-muscular tissues; however, it is likely it differs between different tissues.

As Dp427m, all other dystrophin variants are able to interact with β-dystroglycan to anchor to the sarcolemma. The cell surface subunit of dystroglycan, α-dystroglycan, binds to the ECM proteins such as laminin-2. In such a way, dystrophin-dystroglycans-laminin-2 axis connects the inside and outside of the cell with mechanical assistance given by the subcomplex of sarcoglycans and sarcospan (Fig. 2) [31]. In addition to the mechanical function, dystrophin serves as a scaffold for other proteins controlling reactive oxygen species (ROS) production and involved in calcium handling [32].

DAGC is fundamental but not outlying complex to maintain sarcolemma integrity. The complex composed of transmembrane α7β1 integrin, talin and vinculin, functionally complements and reinforces the connection between ECM and the actin cytoskeleton (Fig. 2) [31]. Integrins, which bind various ligands present in the ECM (e.g. fibronectin, vitronectin, collagen, laminin) are critical regulators of cellular adhesion and mechano-transduction. Both complexes, DAGC and integrin/talin/vinculin together form a larger organizational unit known as costamere, which surrounds the circumference of skeletal and cardiac muscle cells [4, 33]. Submembranous costameres resemble focal adhesion complexes present in most cells (Fig. 3). As such are responsible for the physical communication between the sarcomeric machinery, in particular the Z-disc, the sarcolemma and external environment, transmiting signals in both directions. The Z-line is an important component of the system being an anchor for the sarcomere and a signaling meeting point [34]. Mechanical stress signals received by sarcolemma are transmitted to intracellular pathways that may affect sarcomere assembly, myofibril growth and contraction. The alterations of mechanical load exert changes in myocyte growth to adjust to physiological (heartbeat) and pathological conditions [33, 34].

Finally, costameres govern force transmission maintaining mechanical integrity of the membrane and preventing against contraction-induced damage. A lateral transmission of force, perpendicular to the long axis of the sarcomere, reaching the costameres and transduced to ECM, is the major force vector within striated muscle. Less force (20–30%) is generated by sarcomeres in longitudinal direction, parallel to the long axis of the sarcomere [34].

### DMD mutations—the spot matters

The proper assembly of costamere components poses critical support to maintain the myocyte integrity. As aptly named an Achilles’ heel of Herculean muscle [4], the corruption of costamere machinery can be severely detrimental for the whole muscle. Malfunction of DAGC due to structural or posttranslational defects in one of its constituents, depending on the altered protein, lies at the root of different types of muscular dystrophy (DMD/Becker muscular dystrophy (BMD), sarcoglycanopathies, dystroglycanopathies and others). Among them, DMD reflecting dystrophin deficiency, is the most common and devastating neuromuscular disorder [31].

The majority of *DMD* mutations affect the expression of the muscle isoform [35]. Among them ~ 71% are intragenic deletions that may span one or more exons (usually find in two hotspots: exon 45–55 or exon 3–19),  ~ 11% are exonic duplications and ~ 18% are small mutations (most often nonsense/frameshift types) [2, 36]. Disease severity to a large extent depends on the breakpoints of intragenic mutations, whether or not the reading frame of *DMD* is maintained [37]. Frameshifting mutations most often lead to complete loss of dystrophin and result in DMD phenotype. In case a correct reading frame is maintained, internally truncated protein with preserved function (or semi-functional) may be produced and associated with much milder BMD phenotype. In about 80–90% of cases, such a distinction is adequate: out-of-frame deletions are associated with DMD, while in-frame deletions with BMD or intermediate phenotype [2, 36, 37].

The exceptions to the “reading frame rule” are recognized [36]. Deletions in brain and muscle promoters always give DMD phenotype. In-frame mutations in DMD patients can be associated with partial or total loss of critical protein domains (ABD1 or CRD) resulting in protein dysfunction. On the other hand, frameshift deletions located at the 5' end of *DMD* gene can determine BMD phenotype, with possible involvement of the mechanisms retaining dystrophin expression such as alternative translation initiation and alternative splicing [36].

Several compensatory mechanisms act to mitigate the absence of dystrophin. An increase in α7β1 integrin in *mdx* mice and DMD patients is observed likely to reinforce the connection between the fibers and basal lamina [38]. More strikingly, utrophin, which is normally decreased in postnatal skeletal muscle, accumulates at the sarcolemma and fills the position in cortical cytoskeleton normally occupied by dystrophin, in both dystophic mice models and human condition [30, 39, 40]. Double knockout (dko) mice, with *Dmd* (*mdx* mice) and *Utrn* mutation mirror skeletal muscle pathology of *mdx* mice at ~ 4–5 weeks of age, but then present more severe phenotype, with prominent interstitial fibrosis [41]. Heart pathology of *mdx/Utrn −/− *mice more closely parallels patient condition and first signs of cardiac problems start around 8–10 weeks of age, whereas *mdx* mice evoke milder phenotype detectable from ~ 3 months of age [41, 42]. Accordingly, the lifespan of *mdx* mice is only modestly affected, while is strikingly shortened in *mdx/Utrn −/− *mice, which live only 4–14 weeks (Fig. 6) [41]. The compensatory effect of utrophin was also reported to be relevant to some extent for human condition as its level inversely correlates with DMD severity [43].

## From apparent skeletal muscle weakness to timid symptoms of cardiac malfunction

Clinically DMD manifests as progressive muscle weakness leading to the loss of ambulation at or before age 12. Skeletal muscle symptoms are considered the defining characteristic of DMD. First symptoms are noted in early childhood, usually between the age of 2 and 7 years, and include waddling gait with toe walking, lordotic posture and Gower maneuver indicating weakness of the lower leg muscles and inability to stand without using arms for assistance. Paradoxically, although fiber atrophy decreases the volume of thigh muscles after the age of 7, lower leg muscles (particularly posterior compartment) present characteristic gradual enlargement known as pseudo-hypertrophy [44, 45]. Such a phenomenon occurs due to progressive replacement of muscle fibres by fibrosis and fat combined with slower atrophy than in the thigh muscles. No true hypertrophy of the calves is evidenced in DMD patients that would correspond strictly to increased number and/or size of fibres without accumulation of connective tissue [44]. Deterioration of motor performance starts at 6–8 years when lordosis and scoliosis become evident. At the age of 9–12 years boys are mainly wheelchair bound with preserved upper limbs function. In parallel, respiratory muscle weakness and chest deformity (kyphoscoliosis) predisposes affected boys to respiratory failure [2, 3].

Cardiac problems typically start as latent CM without symptoms evolving into clinically overt cardiac disease with the onset after 10 years of age, to be present in all patients over 18 years of age. According to the report of Nigro et al*.,* before 14 years of age, less than 15% of patients and 57% amongst 18 years old patients with overt cardiac involvement have symptoms [11]. The symptoms of HF such as fatigue, weight loss, vomiting, abdominal pain, inability to tolerate daily activities, dyspnea on exertion or decreased exercise capacity or orthopnea and paroxysmal nocturnal dyspnea, can be either undetected due to musculoskeletal limitations or masked likely due to poor mobility and low exercise capability related to co-existent skeletal muscle weakness or missed due to the use of nocturnal non-invasive mechanical ventilation for sleep [3, 9].

Some degree of intellectual disability is common in DMD patients, however, gradual deterioration has not been reported [39]. An information about the location of mutations within *DMD* gene may help to predict clinical phenotype. Distal *DMD* mutations determining the level of shorter dystrophin isoforms, such as Dp140 and Dp71 predominantly expressed in the brain, are associated with cognitive impairment in DMD [46]. Full-scale IQ scores correlate also with the number of missing isoforms, with the lowest scores associated with the lack of all dystrophin variants [12].

## General DMD diagnosis

At the time of DMD suspicion serum CK is routinely checked as a first step of diagnosis. It rises sharply in early stages of the disease (~ 10–100 fold higher than healthy controls) being related to ongoing muscle damage and the leakage of myofiber contents [47]. CK activity in serum is accompanied by several others muscle-derived proteins, including lactate dehydrogenase (LDH), carbonic anhydrase 3 or myosin light chain 3 [48].

Genetic analysis verifies the presence of *DMD* mutations. Currently, the multiplex ligation-dependent probe amplification (MLPA), is the most commonly used first-line screening method, able to identify exonic deletions and duplications of *DMD* both in male patients and female carriers, conferring an advantage over multiplex polymerase chain reaction (PCR) [49]. Cases with negative MLPA results and/or unrecognized mutation require further evaluation by *DMD* sequencing. Next-generation sequencing (NGS) is increasingly used for detection of large deletions/duplications, point mutations (nonsense, missense, splice site mutation) and small insertions/deletions (indels) [49].

The level or pattern of some useful biomarkers such as prostaglandin D2 (PGD2) and its metabolites [50] or catecholamines and methoxylated amines [51], may serve as additional hints.

Dystrophic muscle sections typically shows not only areas of necrosis, fibrosis, fat and immune cell accumulation but also central nucleation reflecting continual muscle regeneration [52].

## Insights into heart disease in DMD

### Basis—dilated cardiomyopathy as predominant change in the heart

Non-ischemic cardiomyopathies of various pathogenesis are classified based on structural and functional changes for: dilated CM (DCM), hypertrophic CM (HCM), restrictive CM (RCM) and arrhythmogenic CM (previously referred to as arrhythmogenic right ventricular CM) [53]. DCM is common and progressive CM, with poor survival rates [54]. The etiology of DCM is not only heterogenous, most commonly idiopathic and familial/genetic, but also viral and/or immune, alcoholic/toxic roots are described [55]. The available diagnostic tools and invasive and non-invasive testing directly influence categorization. Imprecise evaluation could classify many cases under idiopathic together with those suspected as familial/genetic but with no clear genetic agent discovered [54, 56]. Identification of the specific cause of CM may have prognostic value and contribute to find an adequate therapy for the individual patient [57, 58].

Clinical picture of DCM is characterized by enlargement and dilation of left or both ventricles and impaired contractility (LV ejection fraction, LVEF, less than 40%). The systolic dysfunction occurs in the absence of coronary artery disease, hypertension, valvular disease or congenital heart disease [54, 56]. Most often HF sets in and, without transplant, nearly 50% of patients die within 5 years [54]. Arrhythmias, thromboembolism, and sudden cardiac death (SCD) are typical at any stage [55].

HCM is a genetic disorder (predominantly autosomal dominant pattern) defined by usually asymmetric left and/or right ventricular hypertrophy that involves the interventricular septum and can be associated with diastolic dysfunction [55, 59]. Despite the relatively mild course in most patients, arrhythmias and SCD occur commonly. In contrast to nonspecific histological picture of DCM, HCM is reflected by myocyte hypertrophy, disarray, and interstitial fibrosis [55, 59].

**Fig. 4:**
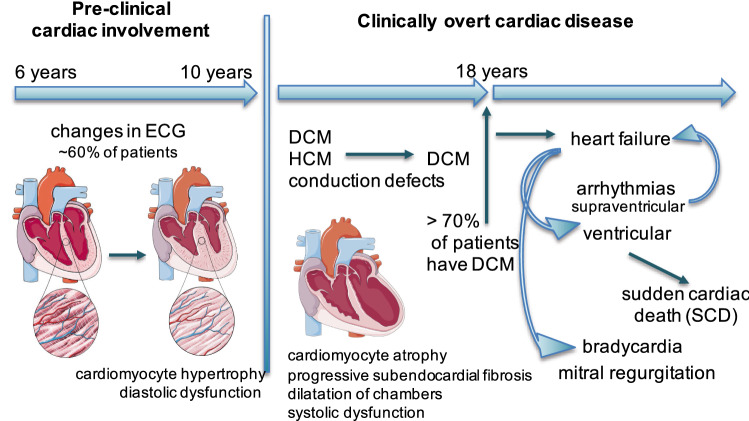
Cardiac involvement in DMD. The first signs of cardiac involvement arising from the absence of dystrophin in cardiomyocytes, may begin by the age of six years and most often develop in the form of dilated cardiomyopathy (DCM), turning into end-stage heart failure (HF) along with associated supraventricular and ventricular arrhythmias. *DCM* dilated cardiomyopathy, *ECG* electrocardiogram, *HCM* hypertrophic cardiomyopathy

In DMD patients at all ages, DCM is more common than HCM or isolated conduction abnormalities. Amongst 328 DMD patients studied by Nigro et al., preclinical signs of cardiac problems such as changes in electrocardiogram (ECG; changes in the duration of the PQ and QT intervals, increased QT:PQ ratio) can be detected already in 25% of patients under 6 years old, are more frequent (59%) up to 10 years, while then begin to drop [11]. At that time, preclinical changes tend to transform into clinically patent/overt cardiac disease (conduction defects, DCM and HCM that may evolve towards DCM) that increases in incidence with age, to be present in all patients over 18 years of age, when more than 70% of patients are diagnosed with DCM (Fig. 4) [11].

CM inevitably progresses towards HF along with associated supraventricular (originating between the sinus node and the AV node) and ventricular (originating below the AV node) arrhythmias. Supraventricular arrhythmias (i.e. sinus tachycardia, the most common type) can potentiate the HF symptoms by decreasing the effective cardiac output. A particular risk is carried by ventricular arrhythmias, which enhance the risk for SCD. In parallel, HF predisposes patients to abnormal heart rhythms. Patients with HF are at high risk of ventricular arrhythmia and SCD. They are also prone to develop symptomatic bradycardia, related to sinus node dysfunction or atrio-ventricular block [60]. In addition, mitral regurgitation and mitral valve prolapse may occur due to LV dilation or fibrosis of papillary muscle (Fig. 4) [9].

### Diagnosis of cardiac involvement—ECG, ECHO, CMRI

An early detection of frequently asymptomatic cardiac malfunction is crucial for adequate clinical management and delay of the onset of overt HF, having direct influence on the outcomes. It is recommended to perform the diagnosis of heart condition at 6 years of age or at the time of DMD onset and repeat it every 2 years until the age of 10, and every year (or often) from 2nd decade of life. Diagnosis should include an examination, electrocardiography (ECG), especially adequate to detect early abnormalities, and non-invasive imaging, echocardiography (ECHO) or cardiac magnetic resonance imaging (CMRI) [9]. Imaging modalities may detect CM even when neither symptoms nor change in cardiac injury biomarkers such as cardiac Tn (cTn) are present. On the other hand, an acute chest pain with accompanying cTn elevation is related to LV dysfunction marking CM progression [61].

Even prior to the development of symptoms, CM can be detected on the basis of ECG changes [11]. Typical abnormalities, associated with corresponding fibrosis, include sinus tachycardia, short PR interval, an increased R/S ratio in the precordial leads with tall R waves, inferolateral Q waves, right axis deviation, and left atrial abnormality [9].

Non-invasive imaging, transthoracic ECHO (TTE) and CMRI with their complementary capabilities, enable deeper investigation of cardiac malfunction. ECHO is relatively inexpensive and rapid method commonly used for evaluation of ventricular function in dystrophic hearts [62]. Typically, it shows LV dilation, regional wall variations and LVEF drop [9]. However, the poor acoustic windows in DMD patients related to altered body habitus, with scoliosis and adiposity of chest wall, can affect the reproducibility and diagnostic utility of the ECHO measurements. Among them, two-dimensional fractional shortening (FS) and 5/6 area-length LV EF are recognized as the most accurate options to detect LV function, having the highest correlation with CMRI [62]. Thus, CMRI able to detect more subtle changes, has become the gold standard for the diagnosis of ventricular structure and function [61–63]. LVEF below 50% can be detected by ECHO already in 9–10 year-old patients (and may drop to 25–30% as disease progresses), whereas CMRI may detect cardiac dysfunction before the measurable LVEF decrease [52]. In a direct comparison to CMRI, ECHO is less accurate in the detection of LV volumes, LVEF and wall motion abnormalities. In addition, the body habitus is not an impediment in case of CMRI. CMRI is able to precisely indicate the border between the blood and endocardium. In combination with late gadolinium enhancement (LGE), it enables tissue characterization and detection of the fibrotic areas, typically associated with early stage of DMD [62, 63]. Of note, progressive fibrosis is strongly correlated with age and LVEF drop [64].

### Location of DMD mutation and isoform pattern vs. cardiac malfunction

The location of *DMD* mutation and related pattern of dystrophin isoform expression may affect the incidence of cardiac involvement. However, not all *DMD* mutations give rise to muscular dystrophy. X-Linked dilated cardiomyopathy (XLDC) relates to dystrophin deficiency and selectively affects heart with no associated muscle weakness or overt muscle pathology. A prominent group of 5’ mutations associated with a severe form of XLDC (5’XLDC) specifically abolish the muscle isoform. The loss of Dp427m is compensated by the brain (predominantly) and Purkinje isoforms in skeletal muscle preventing a myopathy in affected males [23]. However, in the heart, Dp427b cannot replace Dp427m and counteract the ventricular dilatation seen in 5’XLDC patients, as Dp427b expression is confined only to the atria [24, 27].

*DMD* mutations may increase cardiac impairment (involving exon 12, 14–17, 31–42, 45, 48–49 and 79), may be protective for the DMD patients (exon 51–52) or may be neutral for CM (reviewed in [26]). Particularly, mutations affecting specific dystrophin isoforms in the heart (Table 1) may correlate with cardiac involvement [26].

It seems likely that mutations affecting both the full-length and shorter dystrophin isoforms, which retain the ability to bind β-dystroglycan, should cause a more severe CM then those affecting only major isoforms. Unexpectedly, Yamamoto et al*.* suggested that undistorted expression of Dp116 may be detrimental in dystrophic heart [26]. In their study, the retrospective analysis of 181 Japanese DMD patients showed that cardiac dysfunction is less frequent when mutations are found in the region encoding Dp116 comparing to Dp260, Dp140 and Dp71, although no changes in LV dilation were detected [26]. However, such a unique role of Dp116 in the heart over other dystrophin isoforms needs further explanation.

### Pathophysiological mechanisms—troubles begin with membrane instability

The development of clinically overt cardiac disease in DMD is a coordinated effect of several pathophysiological mechanisms in cardiomyocytes involving membrane instability, calcium overload and oxidative stress, inevitably leading to diminished contractile function, cell death pathways, myocardial fibrosis, dysfunction and dilation. A primary defect arising from the lack of dystrophin is membrane fragility. Progressing membrane damage is evidenced by the release of serum biomarkers like CK (most widely used), LDH and others [32, 48, 52].

The calcium homeostasis is critical for the control of myocyte contraction. In healthy cardiomyocytes, membrane depolarization causes small influx of calcium required force excitation and contraction via the L-type calcium channel (LTCC) in transverse tubules (T tubules). Subsequent calcium release from the sarcoplasmic reticulum (SR) is driven by calcium-sensitive ryanodine receptor 2 (RyR2) and is vital for excitation–contraction coupling in the heart [65]. In the cytosol, calcium reacts with the thin filament that comprises actin and regulatory proteins, tropomyosin (Tm) and the cardiac troponin complex (cTn) built from three subunits (T, C, and I) (Fig. 2) [66]. Cardiac troponin T (cTnT) anchors Tn subunits to the filament. cTnC is the direct calcium sensor that undergo conformational changes upon calcium binding. To initiate contraction not only the calcium binding by cTnC is critical but also the interaction between cTnC and cTnI triggering an open cTnC state. When cTnI, the inhibitory unit of the Tn complex, shifts from actin binding toward an interaction with cTnC in the presence of calcium, Tm is free to move, exposing myosin-binding sites on the actin filaments and allowing myosin heads to bind and drive contraction [66].

Under relaxation phase, the calcium is quickly pumped back to SR by numerous pumps in its membrane such as Serca2a, or through the Na + /Ca2 + exchanger (NCX) in the sarcolemma. In addition, physiological stretch leads to microtubule activation of NADPH oxidase 2 (NOX-2), a major source of muscle ROS. In turn, ROS stimulate calcium influx via stretch-activated channels (SACs) [32, 65].

**Fig. 5:**
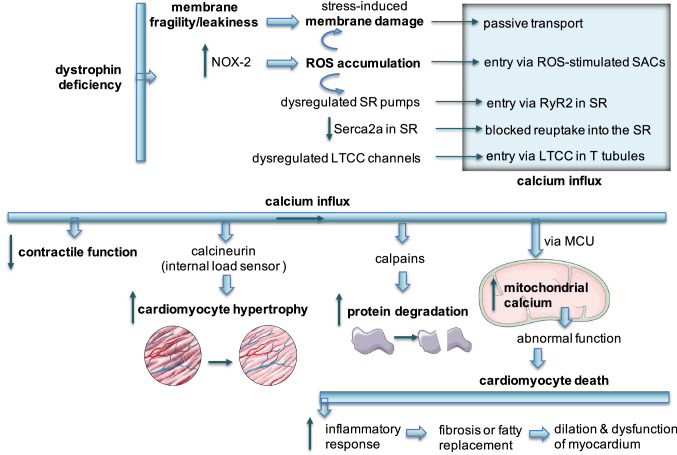
Pathophysiological mechanisms underlying the cardiac involvement in DMD. Clinically patent cardiac disease in DMD is a result of coordinated action of pathophysiological mechanisms in cardiomyocytes with critical role of membrane instability, calcium overload and oxidative stress. Together they inevitably lead to cell death, fibrosis and compromised contractile function. *LTCC* L-type calcium channel, *MCU* mitochondrial uniporter, *NOX* NADPH oxidase, *ROS* reactive oxygen species, *RyR* ryanodine receptor, *SAC* stretch-activated channel, *Serca* sarco/endoplasmic reticulum Ca^2+^-ATPase, *SR* sarcoplasmic reticulum

In dystrophin-deficient cardiomyocytes, sarcolemma instability, related stress-induced membrane damage and so-called micro-tears result in the passive transport of calcium associated with changes in ion gradients. The upregulation of NOX-2 expression and activity drives ROS accumulation enhancing membrane damage and calcium influx through SACs. Changes in the activity of channels such as LTCC (delayed inactivation) add to the inflow of calcium to dystrophic cardiomyocytes [65]. Intracellular calcium level increases more via dysregulated SR pumps. ROS-dependent nitrosylation and oxidation of RyR2s [67] as well as decreased Serca2a expression and/or activity, together contribute to decreased calcium level in SR. Such an aberrant cytosolic calcium load progressively inhibits contractile function of cardiomyocytes [32]. Broader effects are evoked by calcium-responsive enzymes such as calpains, driving protein degradation [68] or calcineurin, inducing hypertrophic growth as a response to alterations in workload (Fig. 5) [69].

The more intracellular calcium, the more mitochondrial calcium is stored. The excess of calcium in the cytosol is uptaken by the mitochondria via mitochondrial uniporter (MCU). At the same time, calcium leakage from mitochondria via mitochondrial sodium–calcium exchanger (NCLX) is blocked by calcium gradient. Calcium overload-associated abnormal function of mitochondria including enhanced ROS production, membrane depolarization, persistent opening of the mitochondrial permeability transition pore (MPTP), decreased ATP production and bioenergetic crisis. It leads to cardiomyocyte death initiating inflammatory response and fibro-fatty replacement, inevitably causing dysfunction of myocardium. Both mitochondrial metabolic alterations and fibrotic scarring appear ahead of overt signs of CM (Fig. 5) [32, 52, 70, 71].

Defects in electrical conduction system are considered critical mechanisms underlying development of arrhythmias related to DCM [72]. Gap junction channels linking neighboring cardiomyocytes via connexin proteins located at the intercalated discs allow for proper ion flow and signal propagation in the heart (Fig. 3). Connexin43 (Cx43), the most abundant cardiac connexin is expressed throughout atrial and ventricular cardiomyocytes. Pathological mislocalization of Cx43 to lateral sides of myocytes related to the uncoupling of hemichannels from intercalated discs has been correlated to DMD arrhythmogenesis in murine models (*mdx* and *mdx/Utrn −/−*). Also, human DMD hearts show lateralized and upregulated Cx43 [72].

On the other hand, in dystrophic mice, a gradual decrease of nNOS at intercalated discs is detected (vs. wild-type mice) [18]. nNOS dysregulation and lower NO production may also add to cardiac malfunction in DMD. Indeed, the heart of *mdx*:nNOSµ* −/− *mice presented an early development of fibrosis (vs. either *mdx* or nNOSµ* −/− *mice) [18]. Accordingly, myocardial expression of nNOS transgene in *mdx m*ice prevented the development of CM [73]. These findings imply that although nNOS localization in cardiomyocytes may not depend on the presence of dystrophin, dystrophin may indirectly affect nNOS activity [18, 70].

## Attempts to defeat heart disease in DMD

Currently, there is no specific treatment for cardiac malfunction in DMD patients, likely due to gaps in understanding of underlying mechanisms that could be related in part to insufficient compatibility of commonly used animal models. At present, treatment is to high extent directed to prevention (to delay the onset of cardiac problems) or the management of symptoms (to improve patient's condition), still leaving the heart problems the major threat of patient’s life.

### Utilized and future treatment options for heart disease in DMD

With the ability of glucocorticoids to reduce inflammation, they constitute a standard of care in DMD. Steroid therapy often starts early, around 2–5 years of age, and undoubtedly evokes beneficial effects on the course of DMD in skeletal muscle i.e. reduces risk of the loss of mobility, the progression of upper limb disease and death [74]. Despite this, there are concerns about systemic side effects associated with classic transcriptional effects of glucocorticoid receptor, including reduced bone density, increased muscle catabolism and obesity [52].

A bunch of DMD patient studies points that glucocorticoids (either prednisone or deflazacort) are likely to be cardioprotective [75–81]. Early initiated steroid therapy may delay development of ventricular dysfunction [78]. The duration of treatment is also inversely correlated with the incidence of CM [75]. The LVEF and FS measures are better and mortality is reduced in steroid-treated patients [76, 80]. Overall, data suggest that early treatment or started upon first signs of subclinical cardiac malfunction, might influence outcomes and prolong survival of affected boys [82]. Nonetheless, the mechanism(s) by which steroids slow the cardiomyopathic process is not yet fully understood.

In addition, some other small molecule-based therapies are used either preventively, before a reduction in LVEF, or under reduced LVEF, and include angiotensin-inhibiting therapies, beta-adrenergic receptor blockers (beta-blockers) or mineralocorticoid receptor antagonists (MRAs). The strategy to target angiotensin II and angiotensin II type 1 receptor, aims to mitigate unfavorable remodeling of myocardium. Angiotensin converting enzyme inhibitors (ACE-Is) (or better tolerated angiotensin receptor blockers (ARBs)) are applied as a standard treatment for patients with HF in general, being used also for dystrophic CM [52]. Data indicate that early treatment with ACE-I (perindopril) retards LV dysfunction and prolongs survival of DMD patients [83, 84]. ACE-I combination with beta-blockers (carvedilol) is also practiced outweighing single treatment, for improvement of LV systolic function and overall outcomes [85]. Although, with their characteristics, beta-blockers are rather used for patients with arrhytmia and symptomatic CM [32].

Utilized treatment and ventilatory support have increased the average age at death and DMD patients can now expect to live into their 4th decade of life [5, 6]. Despite recent progress, cardiac causes of death appear to be increasing and prognoses remain poor [10]. Thus, advanced therapies are often needed and refer to cardiac transplantation and LV assist devices (LVAD). Nowadays, it is clear that to finally defeat this ultimately progressive disease novel therapies have to be developed or verified as clinically relevant. Those should target either primary defect (dystrophin deficiency), secondary consequences (critical steps in pathogenesis of DMD), or both [86]. Although several therapies restoring dystrophin expression have been already conditionally approved, they target only specific *DMD* mutations, by passing nonsense mutations (ataluren) or restoring the reading frame via excision of exon 51 (eteplirsen) and 53 (golodirsen) implying the application only in limited group of patients. In addition, therapeutic effects are debatable, especially in the heart [87].

The strategies of gene therapy have been evolving over the past few years to restore dystrophin expression [88]. In gene augmentation approaches, transduction efficiency is critical problem due to the large size of the dystrophin cDNA. The modulation of the splicing patterns by the use of exon skipping strategy and antisense oligonucleotides, is transient and may give off-target effects. Programmable nucleases (CRISPR-Cas9 system) enable target-site-specific modification, but the way of administration to muscles and off-target mutagenesis needs to be considered. In addition, having in mind stochastic, often extensive genetic changes in *DMD* gene, targeting all them (large deletions or duplication of multiple exons) is a future great challenge. The ex vivo gene therapy approach (i.e. testing cells isolated from patients) sets a rational direction to pre-define the success of gene correction and estimate off-target mutagenesis [88].

### Human DMD heart in vitro—patient on a dish

Arising from cell reprogramming [89] induced pluripotent stem cells (iPSCs) present infinite self-renewal capacity and potential to differentiate into all cell types of adult organism, resembling embryonic stem cells. As such, iPSCs, constituting a new source of patient-specific material, have opened new direction in DMD modeling and treatment [87, 90]. Application of iPSCs in different model systems including three-dimensional (3D) culture approaches enable a better reflection of native tissue (myocardium) [91, 92].

iPSC-derived myocytes provide a unique tool to understand DMD pathology. Indeed, studying the background of DCM, Lin et al*.* used DMD iPSC-derived cardiomyocytes (iPSC-CMs) and reported increased levels of cytosolic Ca^2+^, mitochondria damage and cell apoptosis, a cardiac phenotype also recognized in DMD patients [93]. As revealed by mechanistic studies a mitochondria-mediated network was responsible for increased apoptosis of DMD iPSC-CMs [93]. Such cells showed also electrophysiological abnormalities such as arrhythmias and prolonged duration of action potential [94]. However, the reduced automacity (low spontaneous firing rate) revealed in DMD iPSC-CMs is not characteristic for DMD patients, who usually present enhanced heart rate attributed to elevated sympathetic tone [94]. Further studies are necessary to elucidate whether this reflects different features of not fully mature iPSC-CMs and hence whether the undetected yet clinical changes in young DMD patients might be different from those occurring in symptomatic ones.

With rapidly expanding genetic correction methods, iPSC-derived (cardio)myocytes (with restored dystrophin expression and/or modification of secondary pathophysiological pathways) may constitute new options for DMD treatment by autologous transplantation. In this direction, Young et al*.* used CRISPR/Cas9-mediated deletion (spanning exons 45–55) to re-frame *DMD* gene in DMD patient-derived iPSCs. Such a repair mechanism might cover 60% of mutations seen in DMD patients. Genetically improved DMD iPSC-CMs and skeletal muscle myotubes, produced internally deleted, but functional dystrophin [95]. Furthermore, somatic genome editing resulting in excision of exon 51 in DMDΔ52 patient-derived iPSC-CMs and myoblasts restored expression of re-framed dystrophin, improved cardiomyocyte calcium management and arrhythmogenic susceptibility, and rescued myogenic differentiation [96].

In addition to disease modeling and cell-based therapy approaches, iPSCs can be used as a model for drug discovery. For example, Abujarour et al. have shown that DMD iPSC-derived myotubes have the potential to functionally respond to hypertrophy-inducing factors Wnt7a and IGF-1, which are tested as potential treatments for DMD [97]. On the other hand, a cardiac scaffold-free microtissues (spheroids), comprised of a mix of different cells such as primary cardiomyocytes or iPSC-CMs, fibroblasts and endothelial cells, are increasingly used for drug testing and toxicology. Nonetheless, such complex structures of multiple cell types although mimic well the tissue composition, still provide a practical challenge [91].

Despite a growing body of positive results and applications, the difficulty of recapitulating late-onset disease phenotypes may limit the use of iPSCs. From a clinical perspective, additional studies with experimental animals, still being an invaluable tool for DMD modeling, are crucial [98].

### Large animal models of heart disease in DMD

There is a spectacular collection of animal models of DMD, created to understand dystrophin function and the mechanisms underlying the pathogenesis of DMD, and aiming to verify therapeutic strategies including the proof-of-principle and protocol efficacy and toxicity. Choosing the model that best fits the study question and accurately reflects the human situation is critical but poses a significant challenge [99].

The large animal models (rabbits, cats, dogs, pigs) show the severe phenotype of dystrophin deficiency recapitulating human disease to a large extent, although may present some specific features (Table 2). Typical manifestations of DMD both in humans and dystrophin-deficient animals, include serum CK increase and histopathologic characteristics such as myofiber necrosis, fiber splitting, fiber size variation and hypertrophy [100]. Particularly, dystrophin deficiency in cats, related to as hypertrophic feline muscular dystrophy (HFMD), is manifested as prominent skeletal muscle hypertrophy and calcium deposits, while poor endomysial fibrosis [101]. Among dystrophin-deficient organisms apparent fibrosis as well as other changes progressing with age, such as muscle weakness, akin to human condition, are particularly prominent in dogs. Canine DMD (cDMD) model shows not only histopathological characteristics but also overall clinical course highly similar to DMD patients [99, 100, 102].

**Table 2:** Preclinial and overt cardiac involvement in representative animal models of DMD

Animal	Genotype/ mutation	Lifespan ( ~)		Progression of cardiac involvement					
		Normal	DMD % of deaths	Age ( ~)	Signs	Refs	Age ( ~)	Signs	Refs
MOUSE	*Dmd*^*mdx*^* (B10)* nonsense point mutation in exon 23 of *Dmd*	2 years	22 months	3–4 months	ECG abnormalities (shortened PR interval, prolonged QT interval, andtachycardia) reduced RV EF elevated RV ESV lower LV CO	[42, 110, 123]	9–12 months	Decreased RV SV; increased LV mass contractile dysfunction increased LVEDD, LVESD decreased LV FS and LV EF drop in LV SV	[42, 112, 124]
				6 months	ECG abnormalities (decreased S wave amplitude and S/R ratio) fibrosis	[42, 125]			
	*mdx-Utrn-/-* targeted deletion in *Utrn*		4 weeks – 8 weeks (50%)	5 weeks	Increased HR	[113]	10 weeks	Decreased LV PWd and PWs	[113]
				8–10 weeks	Necrosis & inflammatory cell infiltration prominent interstitial fibrosis ECG abnormalities (decreased S/ R wave ratio) decreased LV EDV decreased RV EF decreased IVSd decreased LV SV and LV CO	[41, 124, 125]	15 weeks	Decreased LV IVSs increased LVIDd increased LV EDV and ESV decreased LV FS and LV EF increased SV	[113]
	*mdx-MyoD-/-* null mutation of *MyoD*		12 months	5 months	LV myocyte hypertrophy increased ventricular diameter	[114]	10–12 months	Necrosisprogressive interstitial fibrosis (primarily in epicardial region of LV)	[114]
	*mdx-Itga7/-* targeted deletion of *Itga7*		< 1 month	3 weeks	Necrotic areas in ventricular walls cardiomyocyte disarray	[126]	–	–	
	*mdx-Cmah-/-* human-like inactivating deletion in *Cmah*		11 months (50%)	3 months	Necrotic foci decreased RV EF decreased RV SV and RV CO decreased LV CO fibrosis at the LV and RV walls	[106, 110]	6 months	Decreased LV EDV decreased LV SV elevated HR decreased LV CO	[110]
	*mdx-mTR*^*G2*^*-/-* deletion of RNA component TERC (mTR) of telomerase, generation 2(G2)		4 months – 18 months (50%)	7 months	ECG abnormalities (prolonged QRS interval) extensive ventricular fibrosis LV wall thinning contractile dysfunction decreased LV FS	[127]	7 months	Increased LV diameter increased LV TAd and LV TAsenhanced markers of heart failure	[127]
RAT	*Dmd*^*mdx*^ targeted deletion in exon 23 of *Dmd*	2 years	Not reported	3 months	Necrosis inflammatory cell infiltration and fibrosisfat tissue infiltration decreased LV EDD	[128]	3 months	LVAW_d_ diastolic dysfunction	[128]
							7 months	Increase in the fibrotic area lower heart weights	
RABBIT	Targeted mutation in exon 51 of *DMD*	6–8 years	2 weeks (20%) – 20 weeks (43%)	4 months	Significant loss of cardiomyocytes cellular infiltration increased interstitial fibrosis	[129]	4 months	Fatty tissue accumulation decreased LV EF and FS	[129]
CAT (HFMD)	Not reported	15–18 years	Not reported	6–7 months	Papillary muscle hypertrophy multiple mineralization foci decrease in echogenicity of LV free wall increasedIVSd chamber dilation	[101, 104]	1 year	Increased LVWTd and LVWTs	[104]
							2 years	LV papillary, free wall, and interventricular foci of mineralization and chronic fibrosis modest thinning of LV free wall and septum reduced contractility mild left atrial dilation and mild biventricular dilation rare clinical signs of heart failure	[100, 104]
				9 months	Increase in echogenicity of endocardium and papillary muscle smaller LVIDd and LVIDs increased IVSs	[104]			
DOG (GRMD)	Splice site point mutation of intron 6 in *DMD* resulting in exon 7 deletion	10–13 years	10 days – 1–3 years, rarely to 6 years	6–7 months	Mineralization foci ECG abnormalities (increased Q/R ratio) increased echogenicity in posterobasal LV wall	[130]	> 2 years ~ 2.5–4 years	ECG abnormalities (sinus arrhythmias, deep Q waves) Increased LV EDV and ESV decreased LV EF and FS fibro-fatty infiltration systolic dysfunction heart failure	[102] [103]
				11–12 months	Foci of myocyte hypercontraction fibrosis decreased LV WTd and LV WTs decreased LVIVSd and IVSs decreased LV FS decreased LV EDDincreased HR	[131, 132]			
PIG	*DMD*Δ52 deletion of exon 52 *in DMD*	15–20 years	1 week (62%) – 3.5 months	< 3.5 months	Decreased LV EF Decreased voltage Amplitude in ventriclesExtensivefibroticareas	[96]	< 3.5 months	Malignant ventricular arrhythmias occurrence of sudden cardiac death no overt signs of heart failure	[96]

Reported signs of heart malfunction are listed when first appeared. CO cardiac output, AWd (AWs) anterior wall thicknessat end diastole (systole), EDD end-diastolic diameter, ESD end-systolic diameter, ESV end-systolic volume, EDV end-diastolic volume, HR heart rate, IDd (IDs) internal diameter at end diastole (systole), IVSd (IVSs) interventricular septal wall thickness at end diastole (systole), SV stroke volume, PWd (PWs) posterior wall thickness during diastole (systole), TAd (TAs) transverse area in diastole (systole), WTd (WTs) LV free wall thickness at end diastole (systole)

Large animal DMD models show significant cardiac involvement (Table 2). Remarkable resemblance to human condition has been recognized in the natural history of GRMD CM. GRMD dogs develop a late-onset, progressive DCM, associated with fibrosis, myocardial strain abnormalities, LV diastolic and systolic dysfunction and LV dilatation. HF may occur from about 45 months of age. Nonetheless, the course of cardiac involvement may vary between different cases of cDMD, as it is in affected boys [103].

The progressive course of cardiac pathology with subendocardial fibrosis typical in DMD patients and GRMD, can be recognized also in feline DMD model [104]. Around 2 years of age, feline dystrophic hearts exhibit LV papillary, free wall, and interventricular foci of mineralization and chronic fibrosis, but also modest thinning of LV free wall and septum and associated impaired contractility and mild left atrial and biventricular dilation [100]. Nonetheless, dystrophic cats only infrequently develop clinical features of HF [104]. Also in dystrophic pigs, despite a reduction of LVEF and extensive fibrosis of LV, there are no overt signs of HF and arrhythmias are considered as dominant cause of death [96]. Nevertheless, it was the pig model (*DMD*Δ52) that was the first clinically relevant large animal model, which in 2020 presented therapeutic success of *DMD* gene editing (deletion of exon 51). CRISPR/Cas9 components administered via adeno-associated viral vectors of serotype 9 (AAV9) resulted inre-framed dystrophin (*DMD*Δ51-52) expression in muscles, amelioration of DMD phenotype and survival [96].

### Rodent (murine) models of heart disease in DMD

**Fig. 6:**
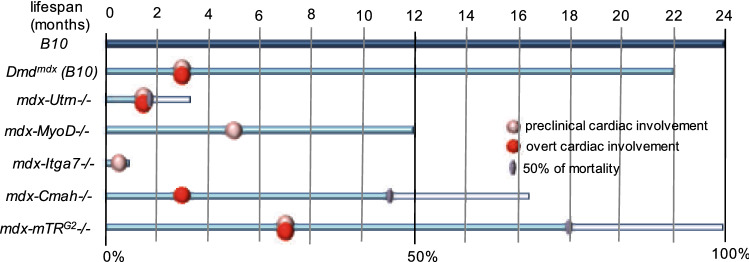
The lifespan of commonly used dystrophic mice in DMD modeling

Despite the relevance and achievements of large animal models, the costs are a major limitation. In regard to that and despite less resemblance, rodents are used commonly to model DMD in humans as they are easy to handle and house, relatively inexpensive and have a short lifespan (Fig. 6), allowing to follow the natural history of the disease and as such should not be underestimated.

#### Mdx mice—genetic variants

The most commonly used DMD model is *mdx* mouse lacking the full-length dystrophin due to a nonsense point mutation (C > T) in exon 23 of *Dmd*. A distinction in size, mechanical loading and lifespan of either dystrophic humans or dogs and *mdx* mice likely implies the severity difference of muscle pathology, with mice showing only mild clinical picture and slightly reduced lifespan (Fig. 6) [99, 105]. Additional genetic differences, like the presence of *Cmah* (cytidine monophosphate-sialic acid hydroxylase) gene, which is silent in humans, may add to the observed differences [106]. Despite that, the *mdx* mice have been invaluable for preclinical studies creating the foundations for building therapeutic strategies in humans.

At 3–6 weeks of age, skeletal muscle of *mdx* mice presents necrosis followed by vigorous regeneration and hypertrophy, with progressive damage (akin to DMD patients) seen solely in diaphragm. At ⁓12 weeks of age, muscle pathology stabilizes, and severe changes such as scoliosis or muscle wasting are not typically present before 15 months of age. Adult muscles usually show only mild necrosis and no fat deposits, while modestly increased fibrosis [99, 107].

The *mdx* murine model is currently complemented by wide range of background and mutation variants. The former include albino, BALB/c, C3H, C57BL, C57BL/10ScSn, DBA/2, FVB or immuno-deficient strains that may vary in dystrophic pathology [99]. C57BL/10ScSn-*mdx* (B10-*mdx*) mice have been widely used in preclinical evaluations, despite less pronounced than in patients with DMD skeletal muscle pathology (except diaphragm) without prominent signs of fibrosis or fat accumulation [108]. The severity of phenotype can be accelerated by crossing B10-*mdx* mice on a DBA/2 J (D2) genetic background and obtaining D2-*mdx* mice with pathologic hallmarks such as prominent necrosis, fibrosis and calcifications [109].

In addition, establishment of chemical variants (cv, with different point mutations) of *mdx* mice such as *mdx*_*2Cv*_, *mdx*_*3Cv*_, *mdx*_*4Cv*_, *mdx*_*5Cv*_, and other dystrophin-deficient lines: *Dup2*, *MD-null*, *Dp71-null*, *mdx52* and *mdx βgeo*, have provided more possibilities to model human DMD, with each strain having some unique features [99]. Moreover, unlike in humans with DMD, additional mutations have been combined with mutation in dystrophin gene, generating several dko mice to accelerate disease progression (Table 2).

#### The heart condition in mdx mice

In contrast to human DMD, RV involvement in *mdx* (B10-*mdx*) model precedes LV dysfunction [42, 110]. CMRI could identify early cardiac abnormalities in the RV like EF drop and elevated end-systolic volume already in 3-month-old animals [42]. Four-month-old *mdx* mice may show ECG abnormalities typical for preclinical stage of human disease, including a shortened PR interval, a prolonged QT interval, and tachycardia [111]. Myocardial fibrosis can be seen from 6 months of age, enhances with age and its severity correlates with cardiac disability. By 9–10 months of age, a DCM may develop, with decreased FS and increased LV end-diastolic and end-systolic diameters and LV mass, followed by a decrease in LV EF at 12 months [42, 108], and a striking increase in interstitial fibrosis in 17-month-old *mdx* mice [112].

The onset and extent of cardiac malfunction may vary depending on genetic background of *mdx* model. In comparison to B10-*mdx* mice, D2-*mdx* strain shows earlier signs of CM, demonstrating myocardial inflammation and calcifications at 7 weeks of age and diminished LV EF and FS starting at ~ 6 months of age [108]. On the other hand, more severe cardiac involvement can be detected in dko mice. *Mdx-Cmah  −/− *model displays pronounced fibrosis of ventricular walls at 12 weeks of age and significant LV involvement by 6 months [110]. The first cardiac changes of *mdx/Utrn −/− *mice also start early, at ~ 8 weeks of age, and gradually evolve in a way comparable to the progression of DCM in DMD patients [41, 113].

Interestingly, although the skeletal muscle-specific bHLH transcription factor MyoD is not expressed in cardiac tissue, *mdx-MyoD −/− *mice exhibit progressive CM, showing that skeletal muscle may determine the development of dystrophic heart pathology [114]. In this regard, microRNA-378a, a crucial regulator of metabolism and muscle biology, highly expressed both in skeletal and cardiac muscle [115, 116], might be of particular importance in DMD. As we have shown, mdx-miR-378a* −/− *mice exhibit better physical performance and attenuated skeletal muscle damage [117]. However, the cardiac involvement in those mice is still to be verified. Nonetheless, it was shown that upregulation of miR-378a-5p together with two others circulating microRNAs (miR-26a and miR-222), is correlated with LGE positivity in DMD patients serving as diagnostic marker for DMD CM [118].

#### Modeling cardiomyopathy in mdx mice

Dystrophic heart presents increased sensitivity to stress. Physical activity, although protective for skeletal muscles, might accelerate the onset of cardiac problems serving as an option to model CM in *mdx* mice. Voluntary physical activity although protective for skeletal muscles, was shown to reduce LV EF and FS and hasten ventricular dilation and fibrosis [119, 120]. Treadmill running with a range of exercise regimens with different inclinations have been also applied [121, 122]. For example, a 10-week protocol (inclination of 7°, twice per week at 15 m/min  – 23 m/min for 60 min) resulted in the activation of intracellular signaling pathways (p38 MAPK, extracellular signal‐regulated kinase 1/2 and calcineurin) and manifestation of dystrophic phenotype in exercised *mdx* hearts [122]. To gradually accelerate cardiac damage, as typical for DMD patients, a 12-week exercise regimen may be used (three times every 2 weeks for 45 min with gradually increased speed up to 12 m/min), moderately increasing workload of the heart. As a result, significant changes in *mdx* hearts are detected including cardiac hypertrophy, fibrosis and reduced contractility [121].

## Conclusions

With advanced DMD management and ventilatory support widely applied from 1990s, there is proportionate increase in cardiac causes of death over those associated with respiratory failure. Clinically detectable CM begins later in life than clinically apparent muscle damage that define characteristic of DMD but is now accepted to be the ultimate threat to patients' lives. Starting by the age of 6, a cascade of events arising from the absence of dystrophin in cardiomyocytes, involving intracellular calcium overload and oxidative stress but also indirect protein dysregulation and/or mislocalization, creates dystrophic heart picture with myocardial dysfunction and irregular electrical conduction. Nonetheless, with no obvious clinical signs of HF undetected/masked by aftermath of skeletal muscle weakness, punctual and precise diagnosis is critical. Ultimately, however, the prognoses for DMD patients are constantly poor with preventive and palliative treatment available. Vast spectrum of therapeutic options remains experimental. Spectacular collection of animal models of DMD, novel patient-specific models originating from cell reprogramming and a range of advanced genetic correction modalities, although related to great expectations, make preclinical study design a great challenge. Wide range of *DMD* mutations only hinder the matter.

## Future directions

Currently, there is no effective treatment for DMD patients with overt cardiac malfunction. Active diagnosis and precise genetic investigation may have prognostic value and may help in personalized disease management, directly influencing the outcomes. Still, novel therapies or combined therapies have to be developed or verified as clinically relevant. Having in mind long lasting difficulties in direct targeting of dystrophin deficiency, strategies addressing the pathophysiological pathways and secondary agents in the heart are essential to halt or reverse the emerging cardiac problems. Ultimately, restoration of dystrophin expression is critical to defeat the primary cause of the disease. Increasing knowledge on the association of the location of *DMD* mutation, the pattern of isoform expression and related after-effects and cardiac involvement could open new possibilities of specific cardiac management.
